# Acute hepatic failure and multi-system organ failure secondary to replacement of the liver with metastatic melanoma

**DOI:** 10.1186/1471-2407-5-67

**Published:** 2005-06-30

**Authors:** Gilaad G Kaplan, Shaun Medlicott, Bruce Culleton, Kevin B Laupland

**Affiliations:** 1 Department of Medicine, University of Calgary and Calgary Health Region, Calgary, Alberta, Canada; 2 Department of Pathology and Laboratory Medicine, University of Calgary, Calgary Health Region, and Calgary Laboratory Services, Calgary, Alberta, Canada; 3 Department of Critical Care Medicine, University of Calgary and Calgary Health Region, Calgary, Alberta, Canada

## Abstract

**Background:**

Metastatic malignant melanoma to the liver resulting in fulminant hepatic failure is a rare occurrence.

**Case presentation:**

A 46 year old man presented to hospital with massive hepatomegaly, elevated liver enzymes and increased lactate three weeks following resection of a malignant melanoma from his shoulder (Clark level 5). Initially stable, he decompensated 24 to 48 hours subsequent to presentation with respiratory failure requiring mechanical ventilation, distributive shock requiring high dose vasopressor infusion, coagulopathy refractory to plasma infusion, progressive rise in liver enzymes and severe metabolic abnormalities including hyperkalemia, acidosis, hyperphosphatemia, hyperuricemia and hypocalcemia. Refractory to aggressive physiologic support he received palliation. Autopsy revealed >80% liver infiltration by metastatic malignant melanoma.

**Conclusion:**

We report a case of fulminant hepatic failure secondary to metastatic malignant melanoma infiltration of the liver.

## Background

Malignant melanoma is a common malignancy that has the potential to metastasize to any site including the liver [1]. However, liver metastases secondary to melanoma rarely result in fulminant hepatic failure [2-5] and do not typically cause significant systemic disease. We cared for a patient with malignant melanoma who developed fulminant acute liver failure and multi-system organ failure.

## Case presentation

A previously healthy 46-year old man on no medications was diagnosed with a Clark Level 5 malignant melanoma on his left shoulder following resection of a mole under local anesthetic. The lesion was subsequently reported as a melanoma and three weeks following his resection, before he could undergo a metastatic work-up, he presented to hospital with nausea, vomiting and right upper quadrant pain. He did not appear toxic, he was afebrile, and physical examination was unremarkable but for a tender right upper quadrant and hepatomegaly. Laboratory investigations demonstrated leukocytosis, modestly elevated liver enzymes, and lactic acidosis. Computed tomography scanning revealed marked hepatomegaly with an extensive infiltrative process and ultrasound demonstrated normal bile ducts. Baseline imaging of his abdomen was planned for staging, however it was not conducted prior to his presentation to hospital.

**Figure 1:**
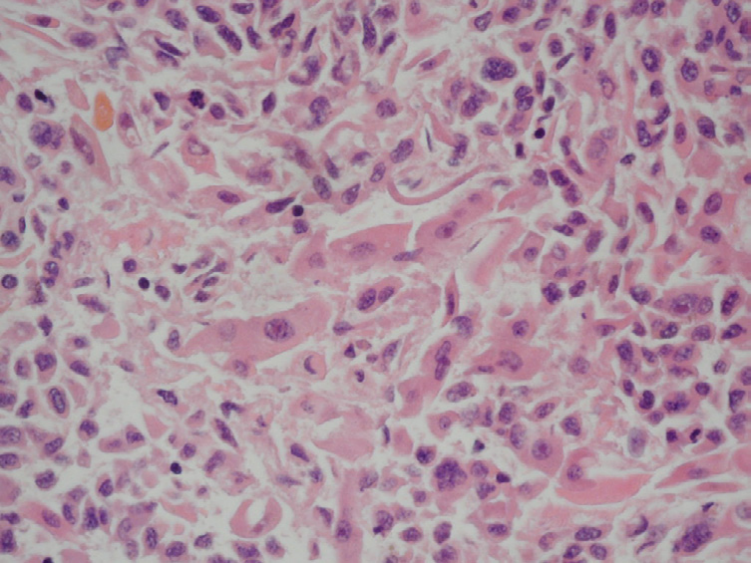
Liver biopsy contains numerous neoplastic elongate cells consistent with malignant melanoma infiltration.

**Figure 2:**
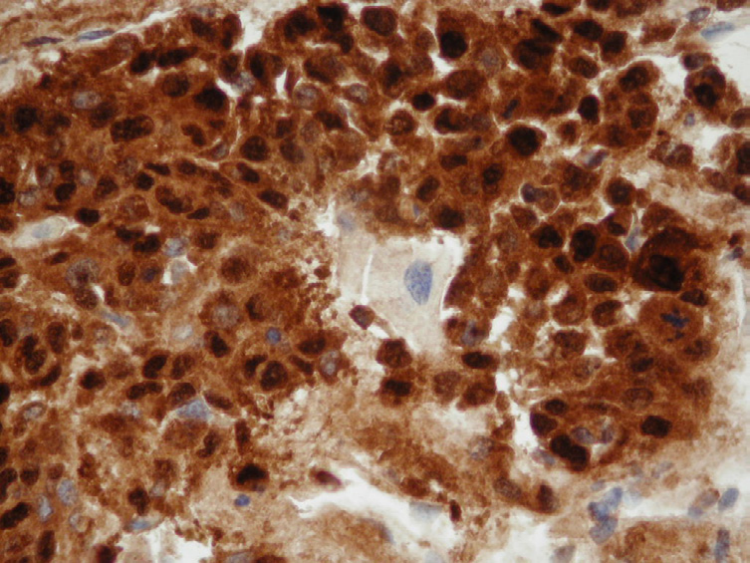
Immunohistochemistry of the liver. S-100 antibody stains malignant melanocytes with a brown chromogen (diaminobenzidine). Rare negative cells (blue due to hematoxylin counterstaining) are intact hepatocytes.

On day two of his admission the patient deteriorated with respiratory failure requiring endotracheal intubation and mechanical ventilation. He had oliguria, and hypotension refractory to fluid resuscitation. Rapidly progressive multi-system organ failure developed with anuria/azotemia requiring continuous renal replacement therapy. Liver failure ensued as evidenced by coagulopathy refractory to plasma infusions and by progressive transaminitis and hyperbilirubinema. He developed distributive shock requiring maximal pressor support. There were significant biochemical derangements including hyperkalemia, acidosis, hyperphosphatemia, hyperuricemia and hypocalcemia. Hemodialysis was commenced to rectify the hyperkalemia and acidosis that was refractory to continuous renal replacement therapy. An aggressive resuscitative and treatment approach was undertaken to allow confirmation that the liver lesions were melanoma and not a potentially treatable infection or lymphoma. An ultrasound guided transjugular biopsy of the liver was performed in the intensive care unit. Based on a frozen section diagnosis of malignant infiltration of the liver, high dose corticosteroid therapy was commenced. The patient continued to deteriorate despite maximal physiologic support and palliative measures were instituted after discussion with the next-of-kin. He succumbed to his illness on day-4 of admission. Autopsy revealed >80% liver infiltration by melanoma (Figure 1), a diagnosis verified by an immunohistochemical phenotype of S-100 and HMB-45 positive cells (Figure 2).

## Conclusion

Our patient had a rare presentation of metastatic melanoma to the liver associated with fulminant liver failure, shock, and multisystem organ failure. Although acute liver failure has been reported subsequent to infiltration of the liver by lymphoma [6], breast [7], gastric [8], and lung cancer [9], there are only rare patients with melanoma associated with fulminant hepatic failure. We conducted an English language search of MEDLINE using the terms hepatic failure and melanoma and only identified four cases of melanoma associated with liver failure [2-5]. The natural history of acute liver failure is typically a rapid progression of liver dysfunction leading to multi-organ failure and subsequently death. Significant rise in lactate dehydrogenase was seen in our patient and this has been previously reported as an ominous sign of acute liver failure [2,5].

The patient's biochemical derangements of severe hyperkalemia, acidosis, hyperphosphatemia, hyperuricemia, hypocalcemia and markedly elevated lactate dehydrogenase most likely represent the natural progression of acute liver failure due to massive hepatic necrosis secondary to tumor burden. However, the patient's biochemical profile can also be seen in tumor lysis syndrome. Tumor lysis syndrome has been reported as a complication of malignant melanoma, however in the context of treatment with chemotherapy [10,11] and corticosteroids [12]. No cases of tumor lysis syndrome have ever been reported to occur spontaneously from malignant melanoma infiltration of the liver. However, spontaneous development of tumor lysis syndrome in other solid tumors without exposure to chemotherapy has been reported in the literature [13-15]. Whether the rapid deterioration of the patient was completely attributable to fulminant hepatic failure or a component of tumor lysis syndrome was involved, this case highlights the potential aggressiveness of malignant melanoma.

The underlying pathophysiology of the acute liver failure in our patient was likely secondary to ischemic injury arising from infiltration of the sinusoids and invasion of hepatic vessels. Hepatic ischemia has been proposed as the mechanism by which lymphoma may cause fulminant hepatic failure [16,17] and is supported by the marked rise in aspartate aminotransferase and lactate dehydrogenase seen in our patient.

In summary, we report an unusual case of fulminant hepatic failure associated with multisystem organ failure resulting from metastatic melanoma replacing the vast majority of the liver. Although rare, our case would portend that malignant melanoma metastasizing to the liver can cause fulminant hepatic failure. Thus, in patients with a history of malignancy who present in acute liver failure clinical suspicion for malignant infiltration of the liver should be considered and ruled out before referral for consideration for liver transplantation.

## Competing interests

The author(s) declare that they have no competing interests.

## Authors' contributions

GK conducted the literature review and wrote the first manuscript draft. SM was the pathologist. BC was the nephrologist and KL was the attending intensivist caring for the patient. All authors contributed to the revisions of and approved the final manuscript.

## Pre-publication history

The pre-publication history for this paper can be accessed here:
